# Prevalence of tick-borne encephalitis virus antibodies in dogs from Denmark

**DOI:** 10.1186/1751-0147-51-56

**Published:** 2009-12-29

**Authors:** Katherine ES Lindhe, Danny S Meldgaard, Per M Jensen, Geoffrey A Houser, Mette Berendt

**Affiliations:** 1 Vangede Small Animal Veterinary Clinic, Plantevej 2, DK-2870 Dyssegård., Denmark; 2 Buddinge Small Animal Veterinary Clinic, Buddinge Hovedgade 222, DK-2880 Bagsværd, Denmark; 3 Department of Agriculture and Ecology, Faculty of Life Sciences, University of Copenhagen, Thorvaldsensvej 40, opg. 2, DK-1871 Frederiksberg C., Denmark; 4 Department of Small Animal Clinical Sciences, Faculty of Life Sciences, University of Copenhagen, Dyrlægevej 16, DK-1870 Frederiksberg C., Denmark

## Abstract

**Background:**

Large regions of central and eastern Europe are recognized as areas where tick-borne encephalitis virus (TBEV) is endemic, including countries neighbouring Denmark. It is therefore timely and relevant to determine if TBEV infections occur in Denmark. This study investigates the presence of antibodies against TBEV in a cross-section of the Danish canine population to assess the level of exposure to TBEV and possibly identify TBEV microfoci in Denmark.

**Methods:**

Blood samples were collected from 125 dogs originating from five regions of Denmark between November 2005 and March 2006. Serum was tested by indirect ELISA. All positive and borderline samples were re-evaluated by neutralisation test (NT).

**Results:**

The prevalence of TBEV serocomplex antibodies was 30% by ELISA and 4.8% by NT (with 100%-neutralising capacity). The island of Bornholm was the only area in Denmark with NT positive samples.

**Conclusions:**

The island of Bornholm is an area with a high risk of encountering TBEV microfoci. The presence of TBEV serocomplex antibodies in many sentinel animals from other parts of Denmark points toward existence of other TBEV microfoci. Discrepancies found between ELISA and NT results stress the importance of careful evaluation of serological tests, when interpreting results.

## Introduction

Tick-borne encephalitis virus (TBEV), a flavivirus, is the cause of the most important arthropod-borne viral disease in central and eastern Europe. It is believed to result in at least 3000 human cases of tick-borne encephalitis annually in Europe [1,2]. TBEV is transmitted to mammals, birds, reptiles and amphibians by ticks of the *Ixodes *family, predominantly by *Ixodes ricinus *[3,4]. The virus causes not only severe meningitis, meningoencephalitis and numerous deaths, but can also induce long-term debilitating complications in patients that survive a severe form of the disease [3,4]. Canine TBE is characterized by lower morbidity, but a higher mortality rate, than human TBE, and dogs are often euthanized because of the severity of their clinical manifestations [4,5]. There is no cure for infection with TBEV and apart from the use of hyperimmunoglobulins in humans over the age of 14 [6], symptomatic therapy is the only means of providing patient support.

Viral existence and the maintenance of TBEV microfoci not only require a microhabitat favorable for *Ixodes *ticks, but suitable hosts and host population dynamics are also important [7,8]. Factors including habitat, seasonal variation and vector-host interactions contribute to the transmission of TBEV. *Ixodes ricinus *exist throughout Denmark and TBEV microfoci have been predicted in many parts of the country, which has raised concern about the establishment of TBEV in areas other than Bornholm [9]. Environmental change to warmer and more humid conditions encourages the spread of tick habitats and establishment of new TBEV microfoci, which pose the threat of new and more abundant infection centers [10].

In Denmark, TBE was first discovered in 1963 on Bornholm, an island of 588 km^2 ^located in the Baltic Sea [11]. At the time when this study was performed, Bornholm was the only location in Denmark where TBEV microfoci had been documented [12,13]. TBEV serocomplex antibodies had, however, been detected in Danish wildlife, indicating that TBE transmission occurred in other areas than Bornholm [14] and, during the summer of 2009, TBEV was found in *Ixodes ricinus *ticks in Northern Zealand [15].

The aim of this study was to examine Danish dogs for serological evidence of infection with TBEV and to estimate the prevalence of TBEV serocomplex antibodies in the animals tested. Furthermore, the study intended to identify the location of potential TBEV risk areas in Denmark as well as possible risk factors associated with a positive titer in dogs. Finally, the use of anti-TBEV enzyme-linked immunosorbent assay (ELISA) in dogs was evaluated for sensitivity and specificity based on the results of the anti-TBEV neutralization test (NT).

## Methods

### Study population and materials

**Figure 1:**
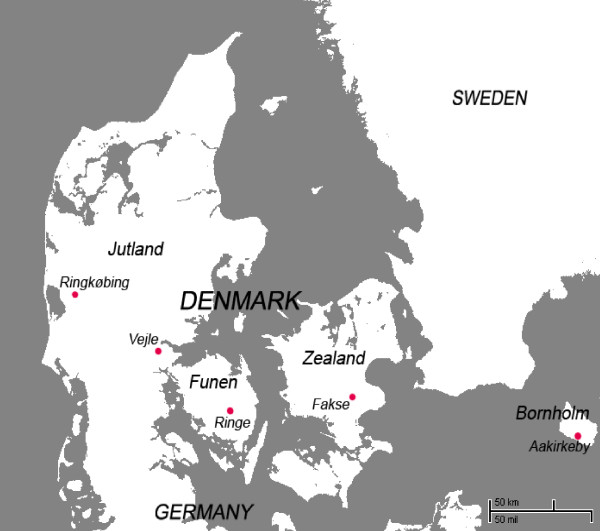
**Geographic distribution of the five veterinary clinics in Denmark that provided canine blood samples**.

The investigation was designed as a cross-sectional study, where dogs were used as sentinel animals and screened for presence of antibodies against TBEV. The study population consisted of clinically healthy dogs. Animals were recruited from five veterinary clinics from different regions of Denmark (Figure 1). Only dogs over the age of 4 years, and weighing more than 15 kg, were included because dogs of this age and size were more likely to have previously visited typical tick habitat such as fields or woodlands. Dogs that had previously travelled to TBE endemic areas outside of Denmark were excluded from the study. For each dog, the following data were collected: place of origin (owner's postal address), sampling month, age, breed, gender and degree of sample haemolysis.

Blood was collected in serum tubes and sent to the Central Laboratory, University of Copenhagen. The samples were centrifuged at 2560 *g *for two minutes (Heraeus Multifuge 1 S-R) and the serum was transferred to small vials, which were kept at -18°C until the time of analysis.

Canine TBEV antibody positive blood samples were obtained from the University of Veterinary Medicine, Vienna, Austria and used as positive controls. Negative control samples were collected from young, small breed, urban-dwelling dogs from Copenhagen that had never travelled.

### Serology

TBEV serocomplex antibodies were detected by a modified indirect ELISA. The ELISA kit, Enzygnost^® ^Anti-TBE virus (IgG, IgM; Dade Behring, Deerfield, IL, USA; User's Manual *March 2005 *p.1-16) intended for use in humans was used in combination with anti-dog IgG conjugate (Bethyl Laboratories, Montgomery, TX, USA) at a dilution of 1:20,000. Canine serum samples were diluted to 1:40. To test the accuracy of the method and the micro titration plates, human negative and positive samples were included in each plate. Human control samples were diluted 1:20, as recommended by the manufacturer. All procedures were performed manually and antibody titer was determined by absorbance of samples in a spectrophotometer with a filter wavelength of 450 nm (Multiscan Ascent V1.24, Thermo Electron Corporation, Waltham, MA, USA). All samples were analysed in duplicate.

ELISA cut-off levels were determined using the manufacturer's recommendation and individual cut-off levels were set for each micro titration plate. Sample results were divided into three groups: negative, positive and borderline, with a cut-off value ≤ absorbance of sample ≤ cut-off + 0.1, as defined by the user's manual. Positive and borderline samples were sent to the Medical University of Vienna, Austria for double-testing by NT, carried out as described by Holzmann and colleagues [16]. Samples with 100% neutralising capacity, measured as = 1:10 in titer level, were considered NT positive. In addition, 5 randomly chosen negative samples were also sent to assess the sensitivity of the ELISA.

### Statistical analyses

Association between selected potential risk factors such as location (postal address), sampling month, age, breed, gender and degree of sample haemolysis and TBEV antibody level was investigated using descriptive and statistical analyses (SAS^® ^9.1 software and Fisher's exact test for statistical significance). The mean, median, standard deviation and variance of titer were determined by Proc Univariate. The extent of interaction and confounding was evaluated in a generalized linear model (Proc Mixed and Proc Genmod). NT results were analyzed using the frequency tables (Proc Freq). Finally, by comparing the results of the ELISA and NT, the specificity and sensitivity of the ELISA was assessed.

## Results

A total of 125 canine blood samples were obtained. Of the 125 samples collected, 38 were positive for TBEV serocomplex antibodies and 19 were categorised as borderline. Figure 1 shows the veterinary clinics where samples were collected.

ELISA positive samples were found in all regions of Denmark included in the study, but a greater number of positive samples originated from Bornholm, where 50% of the ELISA samples were positive, compared to 34%, 30% and 21% on Zealand, Funen and Jutland. No statistically significant correlation was found between location (dog owner's postal address) and a positive titer (*P *= 0.1916 by Fisher's exact test).

Analysis of all ELISA samples showed a seroprevalence of 30.4%. Only six samples (4.8%) were NT positive. Five of the ELISA and NT positive samples were from dogs living on Bornholm. The sixth dog originated from Zealand, but had previously travelled to Bornholm. The five seropositive samples from Bornholm correspond to a regional seroprevalence of 31%. The three ELISA negative samples and two negative controls tested by NT were truly seronegative. The sensitivity of the ELISA was found to be 100% and the specificity 57.6% under the assumption that all 68 ELISA negative samples were negative.

**Figure 2:**
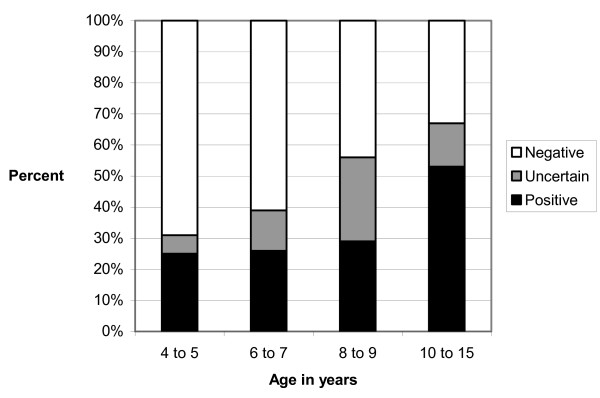
**Distribution of tick-borne encephalitis virus ELISA positive, uncertain and negative canine serum samples grouped according to dog age**.

The age of the dogs ranged from 4 to 15 years with an average of 7.5 years. The average age of ELISA positive dogs was 8.1 years. Although not statistically significant (*P *= 0.0647), the risk of having developed antibodies against TBEV increased with the age of the dog (Figure 2).

Gender had a statistically significant correlation with titer (*P *= 0.0014 by Fisher's exact test). Sixty per cent (39/65) of the female samples were positive or borderline compared to 29% (17/59) of the male samples. One sample of unknown gender was omitted from the statistics. Dog breed and level of sample haemolysis was not statistically correlated with titer. No interaction or confounding bias was indicated between any of the investigated influencing factors (age, month, place of origin, gender). Using the NT data, the only significant influencing factor was place of origin i.e. the owner's postal address (*P *< 0.001 Fisher's exact test).

## Discussion

Our study assessed the prevalence of antibodies against TBEV in a cross-section of Danish dogs to be 4.8% by NT. By means of a modified, indirect ELISA, a seroprevalence of TBEV antibodies was found in 30.4% of the dogs. This prevalence is higher than that found in dogs by a similar method in Norway (16.4%) [17] and that found in humans in TBEV endemic areas in Sweden (12%) [18]. The seroprevalence found in the present study may be higher than that found in Norway because of a more widespread TBEV infection of ticks or because of differences in study population, i.e. greater number of urban dogs in the Norwegian study, or resulting from the possible presence of cross-reactive viruses in Denmark. Seroprevalences of 30-32% have been observed among roe deer (*Capreolus capreolus*) and forest workers on Bornholm [12,13,19]. Similar to this, we found a seroprevalence of 31% in dogs originating from Bornholm.

As argued in other studies of tick-borne diseases, the use of sentinel animals to indicate presence of an infectious agent is rewarding [14,17,20,21]. Dogs are good hosts for ticks because they move through undergrowth and tall grass at the right height for tick nymph and adult attachment. Our findings of antibodies against TBEV in Danish dogs indicate a risk for humans when passing through the same TBEV-infected tick habitats that lead to the infection in dogs.

Although the ELISA titer outcome was not statistically significant, the number of positive samples increased with increasing dog age. Antibodies are known to remain in circulation for extended periods of time and can therefore accumulate, resulting in the highest titers in elderly individuals [22,23]. The average age of positive dogs found by ELISA was 8.1 years, which is consistent with a similar study in Norway [17].

Our study showed a considerable discrepancy between ELISA and NT results. The NT revealed a prevalence of 100% neutralising antibodies in 4.8% of the dogs. This is much lower than that found by ELISA. Although ELISA is known as the method of choice for the detection of TBEV specific antibodies in serum [24], our study shows that use of ELISA as the sole serologic diagnostic method when testing dogs may be insufficient. The sensitivity of the ELISA kit, when used in humans, was reported to be 99.5% and the specificity, to 96.8% [17]. A recent evaluation of the ELISA kit in humans [25] showed sensitivity at 84% and specificity of 78%. In light of the recent discovery of TBEV infected ticks and associated human cases (not evaluated by NT) on the island of Zealand, which had high ELISA anti-TBEV antibody titers, it is reasonable to speculate that these are real TBEV infections that have not lead to the production of neutralizing antibodies detectable by the methods used in this study. The occurrence of ELISA-positive dog sera and lack of NT positive samples from Zealand might be caused by changes in antigen profile during serial passage in the tick population (transovarial transmission [26]), which are reactivated by passage through mammalian hosts. However, at this time we can only speculate about the cause and note that high ELISA titers may have preceded the finding of human cases. Finally, studies on the prevalence of TBEV in Denmark are consistent with the emergence, disappearance and reoccurrence of TBEV microfoci, as has been reported from other parts of Europe in recent years [10,27].

## Conclusion

This study confirms that the island of Bornholm is a TBEV risk area in Denmark. Furthermore, our results suggest that the existence of TBEV microfoci in other parts of Denmark is likely. The study also emphasizes the need for careful evaluation of serological tests when interpreting results in the clinic.

## Abbreviations

TBEV: Tick-borne encephalitis virus; ELISA: Enzyme-linked immunosorbent assay; NT: Neutralisation test

## Competing interests

Baxter Healthcare Corporation has partly financed the data collection but it has had no influence in the design, collection of results, interpretation or preparation of the manuscript at any time. The authors declare that they have no competing interests.

## Authors' contributions

KESL and DSM participated in conceiving the study design, they carried out the ELISA testing and statistical analyses, created the figures and drafted the manuscript. KESL prepared the final manuscript. PMJ supported design of the study, provided technical assistance with the ELISA testing, helped with statistical analysis, and contributed to the manuscript. GH and MB contributed to the study design, participated in coordination and supervision of the project and helped to draft the manuscript. All authors have read and approved the final manuscript.
